# Optoacoustic effect is responsible for laser-induced cochlear responses

**DOI:** 10.1038/srep28141

**Published:** 2016-06-15

**Authors:** N. Kallweit, P. Baumhoff, A. Krueger, N. Tinne, A. Kral, T. Ripken, H. Maier

**Affiliations:** 1 Laser Zentrum Hannover e.V., Hollerithallee 8, 30419 Hannover, Germany; 2 Cluster of Excellence “Hearing4all”, Germany.; 3 Institute of Audioneurotechnology and Dept. of Experimental Otology, ENT Clinics, Hannover Medical School, Feodor-Lynen-Str. 35, 30625 Hannover, Germany

## Abstract

Optical stimulation of the cochlea with laser light has been suggested as an alternative to conventional treatment of sensorineural hearing loss with cochlear implants. The underlying mechanisms are controversially discussed: The stimulation can either be based on a direct excitation of neurons, or it is a result of an optoacoustic pressure wave acting on the basilar membrane. Animal studies comparing the intra-cochlear optical stimulation of hearing and deafened guinea pigs have indicated that the stimulation requires intact hair cells. Therefore, optoacoustic stimulation seems to be the underlying mechanism. The present study investigates optoacoustic characteristics using pulsed laser stimulation for *in vivo* experiments on hearing guinea pigs and pressure measurements in water. As a result, *in vivo* as well as pressure measurements showed corresponding signal shapes. The amplitude of the signal for both measurements depended on the absorption coefficient and on the maximum of the first time-derivative of laser pulse power (velocity of heat deposition). In conclusion, the pressure measurements directly demonstrated that laser light generates acoustic waves, with amplitudes suitable for stimulating the (partially) intact cochlea. These findings corroborate optoacoustic as the basic mechanism of optical intra-cochlear stimulation.

Today, direct electrical stimulation of spiral ganglion neurons (SGNs) using cochlear implants (CIs) is the standard treatment for profound sensorineural hearing loss^1^. The electrical current of CIs targets SGNs and artificially stimulates the auditory nerve. However, alternative excitation methods are being studied for potentially higher spatial delimitation in the cochlea leading to an improved perceptual frequency resolution. It has been shown that neurons cannot only be activated by electrical current^2^, but also by thermal^3^,^4^, mechanical^5^ and optical methods^6^,^7^,^8^,^9^,^10^,^11^,^12^. There are reports of infrared neural stimulation (INS) of the sciatic nerve^9^,^11^, cavernous nerve^7^,^10^, as well as embryo hearts^8^,^12^. Consequently, optical stimulation is being discussed as a potentially higher resolved and artifact-free alternative to conventional CI-treatment.

However, it is not yet clear whether the mechanism of intra-cochlear optical stimulation is based on direct activation of SGNs^13^,^14^,^15^,^16^,^17^,^18^ or whether it is the optoacoustic effect (see materials and methods section, mathematical description of the optoacoustic effect), which deflects the basilar membrane and thus activates the inner hair cells^19^,^20^,^21^,^22^,^23^. This issue is controversially discussed in the pertinent literature.

Based on the theoretical considerations about optoacoustic signal generation we formed the hypothesis that the compound action potential (CAP) response is most likely of optoacoustic nature if it shows similar time dependent characteristics with an onset and an offset signal corresponding to the slope of the laser power (causing a non-zero second time-derivative of temperature, see materials and methods section). However, if it is caused by thermal interaction with membranes or channels of neuronal cells, there should be a dependency on the change of temperature (its first time derivative), which might for example be given by constant laser power during the pulse (flat phase within the pulse shape in contrast to onset and offset) heating the target volume. Previous studies have reported on photothermally induced membrane currents in neurons of C. elegans^24^, in HEK-293T cells^14^,^20^ and spiral ganglion neurons^20^. However, in none of these studies a generation of action potentials by the photothermal impact of laser radiation could be demonstrated. As a possible approach to reach the threshold for photothermally evoked action potentials suggested the use of sensitivity enhancing mediators such as gold nanoparticles^25^.

The laser-induced acoustic wave leads to a traveling wave along the basilar membrane and subsequent excitation of the auditory nerve. Most publications about optical stimulation of the cochlea were performed with pulsed lasers in the near-infrared (NIR) range^16^,^18^,^23^,^26^,^27^,^28^. A previous study has shown a wavelength dependency of evoked potentials between 420 nm up to 2150 nm^22^. The CAP amplitude recorded from hearing guinea pigs increased with increasing absorption coefficient until a reversal point. The negative correlation commences with an absorption coefficient of water above 5.5 cm^−1^ at a wavelength of 1370 nm^22^. Beyond this reversal point, the CAP response decreased with a higher absorption coefficient (negative correlation). The main absorbers were hemoglobin (visible light) and water (near infrared regime)^22^. The characteristic dependency of CAP response amplitude of hearing guinea pigs on the absorption coefficient and laser parameters and the lack of auditory responses of completely deaf animals led to the assumption of an optoacoustic stimulation mechanism. This hypothesis is also supported by observation of basilar membrane vibrations caused by laser pulses^29^,^30^,^31^. The reversal effect in CAP response at higher absorption coefficients remained to be clarified in detail. One possible explanation discussed in *Schultz et al.*^22^ is that the form of the heated volume functioning as the source term for the optoacoustic effect might change from a far reaching linear/elliptic type to a short ranging acoustic point source confined to the direct vicinity of the fiber tip due to the reduced light penetration depth. At a fixed distance from the tip this geometry change would lead to the reversal CAP dependency on absorption coefficient. Therefore, in this study we looked for this characteristic feature in the pressure signal itself excluding physiological reasons for the reversal.

The distinction between optoacoustic hair cell stimulation and other mechanisms of neural stimulation (INS) is decisive for future applications. Hence, our investigation aims to clarify the underlying mechanism of optical stimulation of the cochlea. The objective of this study was to test the hypothesis that CAPs following pulsed laser stimulation in hearing animals are evoked by the optoacoustic effect. In order to verify (or falsify) this hypothesis we searched for the typical features of optoacoustic signals described above by comparison of *in vivo* CAP measurements with pressure measurements using the same laser parameters.

## Results

The results are structured in different laser parameter regimes and the corresponding experimental settings (see also materials and methods): (1) constant laser pulse energy of 6 μJ with varying pulse duration between 5 ns up to 200 μs (hence, varying pulse peak power) in *in vivo* guinea pig experiments (CAP) and physically in water as sound pressure measured with a hydrophone, (2) constant laser pulse peak power of 150 mW with varying pulse durations between 10 μs up to 10 ms (hence, varying pulse energy) *in vivo* and in water, (3) varying wavelength between 845 nm up to 2100 nm with water absorption (hence, varying absorption coefficient) in water, and (4) varying absorption coefficient between 1.34 cm^−1^ up to 77 cm^−1^ at constant wavelength of 1300 nm (via rising concentration of black India ink as an absorber) in water.

### (1) Constant pulse energy

**Figure 1:**
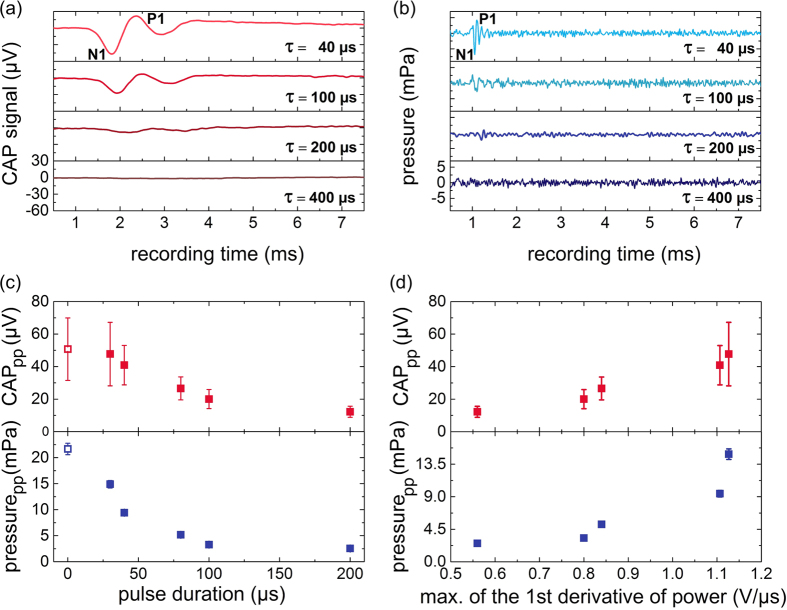
CAP signal (**a**) and pressure amplitude (**b**) for constant pulse energy of 6 μJ of selected pulse durations. The laser pulse delay was 1 ms and N1 is the first negative peak and P1 the first positive peak. (**c**) CAP amplitude (top, n = 8) and pressure amplitude (bottom, n = 5) measured using a hydrophone as a function of laser pulse duration (from N1 to P1). The shortest pulses (empty squares) were generated with the Ekspla laser at 5 ns pulse duration in stress confinement, the other with the Capella laser in thermal confinement. (**d**) The CAP (top) and pressure (bottom) amplitude depending on the maximum of the first time derivative of power.

For constant pulse energy of 6 μJ the amplitude of CAP signals (Fig. 1a, N1 to P1) decreased with increasing pulse duration (Fig. 1c, top). The amplitudes for the laser pulse duration of 5 ns (Ekspla laser) and for the 30 μs laser pulse (Capella laser) were similar. Although the laser pulse of 5 ns met the condition of stress and the laser pulse of 30 μs of thermal confinement only, there was no significant difference between both confinements regarding the resulting CAP amplitude. The amplitude decreased continuously with decreasing pulse peak power (Fig. 1c, top). For pulse energy of 6 μJ no sound pressure or CAP signals were detectable for pulse durations longer than or equal to 400 μs (data not shown). For hydrophone measurements the same constant pulse energy of 6 μJ and optical fiber (105 μm core diameter) were used (see materials and methods). With longer laser pulse durations and the constant pulse energy, the pressure amplitude decreased similar to the CAP amplitude (Fig. 1b,c, bottom). Both signal amplitudes did not depend on the pulse energy and decreased with pulse duration. The CAP amplitude (Fig. 1d, top) as well as the pressure amplitude (Fig. 1d, bottom) increased with an increasing maximum of the first derivative of the laser pulse power (see materials and methods, stimulating lasers). Because of the dependency on the first derivative of power these results strengthen the hypothesis of an optoacoustic behind the CAPs.

### (2) Constant pulse peak power

**Figure 2:**
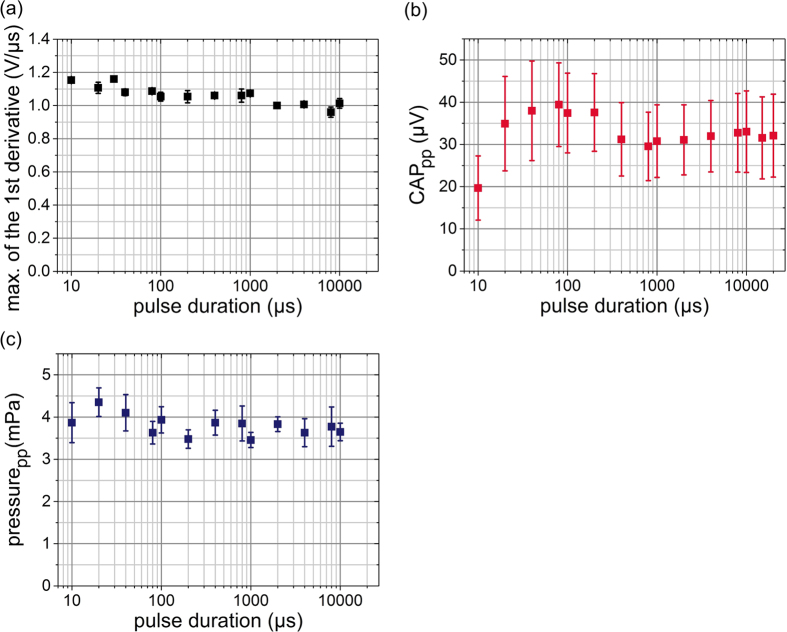
(**a**) The maximum of the first derivative of the detector output for all pulse durations was nearly constant for a constant pulse peak power. (**b**) CAP (n = 8) and (**c**) pressure amplitude measured using a hydrophone (n = 5) as a function of pulse duration for constant pulse peak power of 150 mW. Between 10 μs to 20 ms pulse duration only the thermal confinement condition applied.

In the previous section the maximum of first derivative of power decreased with increasing pulse duration, because the integral energy of the pulse was kept constant. In order to reach a more constant 1st derivative of power the pulse peak power has to be kept at a constant level while varying the integral pulse energy by prolonging the pulse. The pulse peak power was kept constant at 150 mW and the pulse energy was varied with the pulse duration. In this parameter regime the condition of stress confinement was not possible with a power of 150 mW due to the limitations of the laser. The maximum of the first derivative of laser pulse power was almost constant for the fixed pulse peak power (Fig. 2a). Contrary to the assumption that CAP amplitude strictly follows the first derivative of laser pulse power calculated by the recorded photodiode signal, the CAP amplitude increased up to laser pulse durations of 80 μs (Fig. 2b). At that point, the amplitude reached a maximum and decreased towards longer pulse durations of 400 μs. From 400 μs on the amplitude stayed almost constant with increasing pulse duration. However, except for very short pulse durations, variations remained moderate (within approx. 30% below the maximum). In contrast to the *in vivo* data, the pressure amplitude measured in the water basin was almost constant for a fixed pulse peak power (Fig. 2c), similar to the first derivative of laser pulse power (Fig. 2a).

**Figure 3:**
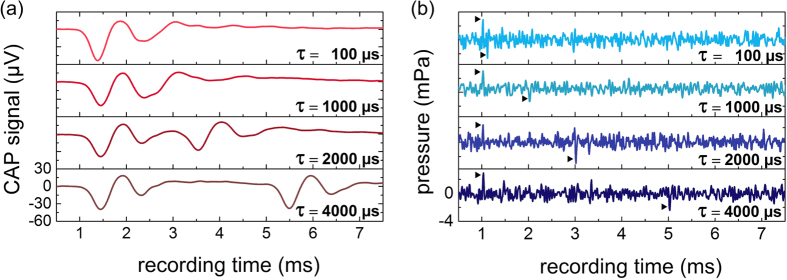
(**a**) Selected CAPs of a representative example showed an onset and offset response for pulse durations longer than 1 ms. (**b**) In hydrophone measurements pressure amplitudes always showed an onset and offset response (arrows) statistically significant above the noise floor (p < 0.001). The laser pulse delay was 1 ms for both measurements.

For pulse durations longer than 1 ms two separate CAP signals were observed (Fig. 3a). The interval of these signals corresponded exactly to the applied laser pulse duration. For pulse durations between 10 μs up to 1 ms separate onset and offset responses were not evident and only a single CAP signal was detectable (Fig. 3a, 100 μs and 1000 μs). In hydrophone measurements, separate pressure pulses were apparent at all pulse durations applied (Fig. 3b). The general correlation of the sound pressure amplitude as well as CAP amplitude with the first derivative of power and the second observation of separate CAP signals (Fig. 3a) and pressure peaks (Fig. 3b) speaks in favor of the hypothesis that optoacoustic is the mechanism behind the CAP signals.

### (3) Wavelength and (4) absorption coefficient study

**Figure 4:**
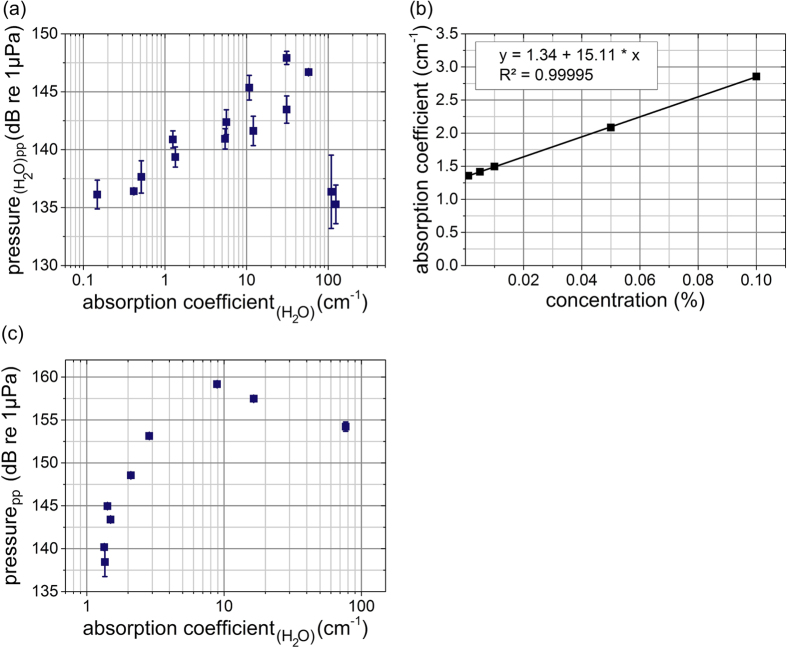
(**a**) Peak pressure amplitude (n = 3) of hydrophone recording inside a water-filled cylinder as a function of absorption coefficient of water^32^,^33^. The pressure amplitude and the absorption coefficient showed a positive correlation up to 57.5 cm^−1^; but not above. (**b**) India ink concentration and the associated absorption coefficient: The values were measured using a spectrometer and a linear correlation was performed to determine the remaining ones (R^2^ = 0.99995). (**c**) Pressure signal (n = 2) for different concentrations of India ink and, thus, different absorption coefficients for a constant wavelength of 1300 nm and pulse energy of 6 μJ.

A positive correlation between optoacoustic pressure amplitude and wavelength-dependent absorption was found up to an absorption coefficient of approximately 57.5 cm^−1^ (Fig. 4a). Used wavelengths and the associated absorption coefficients for water can be found in Supplementary Table S1. Measurements at absorption coefficients beyond 124 cm^−1^ were technically not possible. At wavelengths of 845 nm or lower (μ_a_ ≤ 0.039 cm^−1^) pressure signals in water were not detectable (data not shown).

For measurements with black India ink as an absorber dispersed in water, the wavelength was kept constant at 1300 nm and laser pulse energy was set to 6 μJ. In Fig. 4b the absorption coefficients for different ink concentrations at this wavelength and the linear correlation (R^2^ = 0.99995) are depicted. For a wavelength of 1300 nm the absorption coefficient of pure water is 1.34 cm^−1^ 32,33. Therefore, it is below the reversal point of the wavelength study mentioned before (Fig. 4a, 57.5 cm^−1^). The transmission of laser light for the concentrations >0.1% were outside the measurement range of the spectrometer and were linear extrapolated (Fig. 4b). The results of the pressure measurements for the India ink solutions are shown in Fig. 4c. At the beginning, the pressure amplitude increases with increasing absorption coefficient. However, around a reversal point of μ_a_ = 8.89 cm^−1^ the pressure amplitude begins to decrease again. In principle this finding reproduces the behavior of CAP signal found in *Schultz et al.*^22^ in the pressure signal.

## Discussion

This study investigates the auditory responses of hearing guinea pigs and sets the responses into relation with measured pressure amplitudes generated by laser pulses in water in the same parametric range. Therefore, our results can be regarded as verification of the optoacoustic mechanism underlying intra-cochlear optical stimulation. The assumption of an optoacoustic mechanism alone was sufficient to explain a CAP response of the cochlea with intact hair cells and was also sufficient to explain the absence of CAP in acutely deafened animals with global hearing loss^22^.

The results present strong evidence for optoacoustic as a dominant mechanism of intra-cochlear excitation in animals with residual hearing. This casts doubt on findings of direct optical stimulation of SGNs in experiments performed in normal hearing animals or for those with some degree of residual hearing^15^,^16^,^18^,^34^,^35^. Furthermore, care has to be taken to prevent any form of conductive hearing loss, e.g. by damaging the ossicular chain during surgical procedures, because animals with sensory hearing loss and such a condition might appear to be deaf in conventional acoustic stimulation but may still well respond to intra-cochlear optoacoustic stimulation. Overall, the pressure amplitude induced by laser light was sufficiently high (130–160 dB re 1 μPa in water, Fig. 4a,c) to even stimulate animals with severe hearing loss. The results show that comparatively low energy can produce high pressures in liquid media. An *in vitro* study with SGNs has shown that action potentials cannot be evoked by nanosecond laser pulses over a broad wavelength range from 420 nm up to 1950 nm^20^. In this study, no qualitative difference was observed between *in vivo* responses evoked by nanosecond pulses compared to microsecond pulses or even pulses lasting several milliseconds. Rather two separate responses for longer pulses *in vivo* corresponding to the separate pressure pulses seen in the pressure measurements could be observed.

The pressure at the onset of the signal depended on the first derivative of power. The pressure amplitude at the end of the laser pulse did not depend exclusively on the maximum of the first derivative as also observed by *Gao et al.*^36^. The findings of the onset and offset signal for constant pulse peak power (Fig. 3b) strongly support the assumption that optoacoustic is the basic mechanism for optical cochlear stimulation. The time between the beginning and the end of the response signal are equal to the pulse duration. In accordance with the differential equation governing optoacoustic signal generation (see materials and methods) there is no pressure wave created during the constant pulse power plateau of the laser pulse. The photothermal heating, which is constant in time, cannot generate a pressure wave (equation 6). The fact that in CAP recordings separate onset and offset responses could only be seen for pulses longer than 1 ms, in contrast to two separate pressure pulses discernible in all pressure measurements, can be explained by the prolonged integration time of the compound auditory nerve response acting as a low pass filter. The second CAP signal of the 1,000 μs laser pulse or shorter was superimposed by the first CAP signal of the laser pulse onset (Fig. 3a). Such a long integration time does not exist for the hydrophone with a linear response and an upper frequency limit of 200 kHz. Here, the resolution of the onset and offset signal in pressure measurements was limited by the sampling frequency, which was in our experiments 102 kHz. The pressure showed a difference in polarity between on- and offset signal. The reason for this behavior could be explained with equation (4). This characteristic cannot be observed for CAP signals that are non-linearly rectifying and with a signal form that is maintained. The pressure amplitude at the beginning of a laser pulse of constant pulse peak power and constant maximum of the first derivative is maintained independently of the pulse duration and supports the hypothesis of the optoacoustic effect. The observed slight increase of CAP amplitude and the maximum around 80 μs can be sufficiently explained by the physiology of the guinea pig auditory system. The overall best frequency of the hearing threshold of guinea pigs is between 8 kHz and 12 kHz^37^. The pressure pulses at the beginning and the end of each optical pulse can be considered to be an excitation period. A period of 80 μs corresponds to a frequency of 12.5 kHz and a period of 125 μs to a frequency of 8 kHz. In this frequency range the guinea pig cochlea is most sensitive and thus the CAP amplitudes for a given pressure intensity are highest. For pulse durations longer than or equal to 400 μs the CAP amplitude was almost constant, similar to the pressure amplitude, because the pressure peak of the beginning and the end of the laser pulse were thus far apart, being processed as separate stimuli. The period is consequently less crucial and the rarefaction as well as condensation of the pressure wave was separated.

The optical stimulation in stress confinement, thermal confinement as well as outside of both confinements is based on the optoacoustic mechanism. Our data confirm previous results on optoacoustic effects induced by laser light in thermal confinement^23^. There is no difference in thermal (τ ≤ 4.9 ms) and no confinement (τ > 4.9 ms) for constant pulse peak power. The hypothesis by *Schultz et al.*^22^ that hemoglobin is the main absorber for wavelengths between 420 nm and 845 nm is corroborated by the fact that the hydrophone measurements never showed any pressure signals in water (data not shown). In an earlier publication of *Schultz et al.*^22^ a reversal point where the positive correlation in *in vivo* CAP measurements failed (5.5 cm^−1^) was discovered. In this study, the reversal point with water was approximately at 57.5 cm^−1^ (Fig. 4a) and with India Ink at 8.89 cm^−1^ (Fig. 4c). Obviously higher absorption beyond a distinct turning point leads to an adverse effect on optoacoustic signal amplitude. In optoacoustic pressure measurements in water the point of correlation reversal was shifted towards higher absorption coefficients by a factor of 10 as the *in vivo* measurements. Despite this shift the remarkable common aspect of these measurements remains the reversal point: A higher absorption coefficient does not always lead to pressure pulses of higher amplitude and–by inference–therefore an increase in optoacoustically evoked CAP amplitude. A possible explanation is that high absorption leads to a longitudinal confinement of the optoacoustic source geometry. The shape is altered from a line source along the laser beam to a point source close to the fiber tip. The increasing absorption coefficient leads to a decreasing penetration depth. For strong absorption (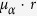 ≫ 1^38^), the heating zone forms a thin disk at the fiber tip. For weak absorption (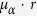 ≪ 1^38^), the heating zone is an extended cylinder along the optical axis. The values of 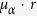 at the reversal point are 0.3 and 0.05 for water absorption (varied by wavelength) and India ink absorption (varied by concentration), respectively. Both values are below 1, but not much smaller than 1. They are within a transition between disk and cylinder geometry. Consequently, the reversal points can be associated with a change of heating zone geometry. Attenuation of the induced acoustic wave with distance to the source is higher for the point source geometry than for the line source geometry. Further research on the acoustic wave geometry is necessary to analyze the drop in evoked sound pressure at high absorption in detail.

The distance between fiber and hydrophone can be adjusted reproducibly. In contrast, the distance between fiber and basilar membrane can only be approximated during the experiments. For this reason and because of the different hearing thresholds of the animals, the standard deviation is greater than the deviation of the pressure measurements. Contrary to other optoacoustic measurements at the fiber tip^39^,^40^, the laser pulse energy of our experiments was too low to generate a cavitation bubble at the distal fiber tip. Consequently, vapor bubble formation could not have affected our results.

The interaction mechanism between the pulsed laser light and the SGNs during INS is still unknown^41^. The deposited energy will of course always produce heat and if the first derivative of power is high enough produce also an optoacoustic pressure wave which may also act on SGN. Whether optoacoustic is the only possible mechanism to optically stimulate primary auditory structures is not solved sufficiently, as we cannot rule out the involvement of a photothermal effect. The unambiguous proof of photothermally evoked acion potentials in auditory SGNs is still open. The process may have a high energy or power threshold to be overcome. The report of *Carvalho-de-Souza et al.*^25^ has shown that gold nanoparticles must be used to enhance the photothermal effect and depolarize the membrane to produce an action potential photothermally^25^. However, the data presented here is unambiguous in its finding that the major factor generating cochlear responses in residually-hearing ears is the optoacoustic effect. If a photothermally induced temperature rise can directly induce an action potential at all remains to be demonstrated on isolated SGNs directly (which was unsuccessful in *Rettenmaier et al.*^20^) or using completely deaf animals (which was unsuccessful in *Schultz et al.*^22^).

## Conclusion

The similarity of the results of *in vivo* and pressure measurements strongly supports the hypothesis of optoacoustic as the main stimulation mechanism. For the optical cochlear stimulation with laser light in stress and thermal confinement functional hair cells are required. The amplitude of the evoked pressure pulses as well as CAP amplitude in hearing guinea pigs mainly depends on the laser pulse shape and particularly on the time derivative of pulse power, either positive at the pulse onset or negative at the pulse’s end, and not on the energy or duration of constant power within the pulse. Higher absorption coefficients cannot automatically evoke higher pressure or greater responses respectively.

## Materials and Methods

### Mathematical description of the optoacoustic effect

The optoacoustic effect was discovered by Bell in 1880^42^ using modulated light. The mechanisms using pulsed lasers works as follows: A medium, here the perilymph inside the *scala tympani*, is irradiated with pulsed laser light. Depending on the wavelength of the laser light in use the photons are absorbed by resonance with transitions of electronic energy levels (typically visible wavelength) or by resonance with transitions in the vibrational energy levels (infrared wavelength) within the molecules. Here, water was the dominant component of all fluids in the beam path like perilymph and endolymph^43^ and therefore is the main absorbing constituent in the near infrared wavelength region. The irradiated volume will have a higher temperature than the surrounding medium. This heating can be expressed as a heating function (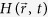), which is defined as the absorbed energy converted into thermal (internal) energy per unit volume and per unit time^44^. The condition is called thermal confinement if the energy of the laser pulse is deposited into the volume much faster than the heat flux to the surrounding volume by heat conduction^39^. It depends on the laser pulse parameters applied, the laser beam dimensions (delivering fiber diameter), the absorption coefficient and the thermal conduction coefficient. The maximum laser pulse duration (full width of half maximum assuming a Gaussian shaped time course of laser power) for thermal confinement τ_th_ is defined as being shorter than the time for heat flux of absorbed energy inside the heated region^39^:


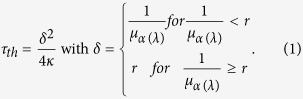


Here, δ is the smallest dimension of heated volume, which is either the axial penetration depth of light into the medium or the radius of the optical fiber r, *κ* is the thermal diffusivity of the sample and μ_α(λ)_ is the absorption coefficient depending on the wavelength.

Analogous to thermal confinement, stress confinement is a condition, in which the pulse duration is shorter than the time necessary for the stress wave to pass through the heated region (τ_p_)^39^:


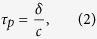


whereas c is the speed of sound and δ is the smallest dimension as defined before. If laser pulses fulfill the condition of stress confinement, the energy will be deposited instantaneously into the interaction volume. Given the condition of stress confinement, the relationship between pressure and heat in an inviscid and acoustically homogenous medium can be expressed as the differential equation^44^:


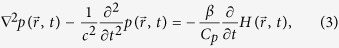


with p being the pressure, c the speed of sound, β the thermal expansion coefficient and C_p_ the specific heat capacity at constant pressure. The initial optoacoustic pressure can be thought of as a delta peak and the solution of the differential equation can be expressed as^44^:


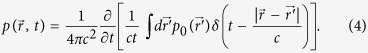


In this case the pulse duration is shorter than the heat flow and the travel time of the stress wave propagating out of the irradiated volume. The absorbed energy is converted into one stress pulse with the initial optoacoustic pressure:


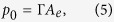


where A_e_ is the specific or volumetric optical absorption (in joules per volume, optical energy deposition density) and Γ is the Grueneisen coefficient. Note that in equation (4) the time derivative is taken from the delta function so 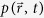 has a front side with positive pressure (condensation) and a back side with negative pressure (rarefaction)^44^.

As soon as the initial optoacoustic pressure cannot be approximated by a delta peak (no stress confinement, but still thermal confinement) the laser pulse width and pulse form in time become important and the actual time dependence of the heating plays a role^44^:


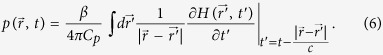


Note, that the pressure amplitude depends on the velocity of heat deposition (slope in time), meaning a dependency of the second derivative of temperature in time^44^. The first derivative of temperature is proportional to the absorbed power per volume. Heating which is constant in time does not result in a pressure wave.

If heat deposition decreases (negative slope in time) there will be an optoacoustic signal with reversed sign. The heating follows the fluence rate of the laser pulse in time, having a positive slope at the pulse onset and a negative slope at the pulse end. The other consequence is that only fast changes create measurable pressure signals and constant power level energy influx will not generate detectable acoustic signals. An optoacoustic signal will be produced only if the photon influx changes in time. If the derivative is high at the start and end of such flat top pulses two optoacoustic signals will be generated: one at the beginning and one with reversed sign at the end.

### *In vivo* study

**Figure 5:**
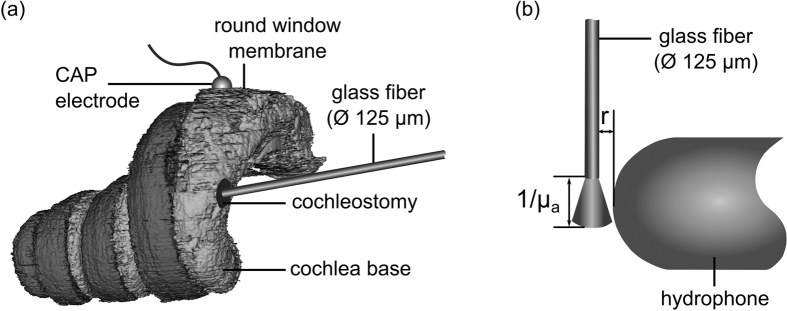
(**a**) Segmented cochlea of a SLOT (scanning laser optical tomography) dataset with a cochleostomy inside the basal turn. An optical glass fiber was placed inside *scala tympani* and a CAP electrode was located at the round window membrane (RWM) for recording auditory responses as schematically shown. (**b**) Schematic illustration of the hydrophone and optical fiber position inside water as a sample medium for pressure measurements (not to scale).

All *in vivo* experiments have been performed in accordance to the animal welfare guidelines of Germany and the European Union. All experimental procedures were approved by the German state authorities and the animal welfare officer of the research facility. The experiments were performed on eight normal hearing Dunkin Hartley guinea pigs (280–755 g, Charles River) of either sex. The initial anesthesia preparation contained ketamine (50 mg/kg), xylazine (10 mg/kg) and atropine sulfate (0.1 mg/kg). The injection volume of the initial dose was adjusted to bodyweight. The absence of paw withdrawal reflex and eyelid closure reflex was tested at regular intervals during the experiment to verify adequate anesthesia depth. The animal was maintained in an areflexic state by injections of 20–30% of the initial ketamine and xylazine doses without further atropine supplementation at intervals of ~45 min. The body core temperature of the animals was measured by a rectal temperature probe and was kept between 37.5 °C and 38.5 °C via heating pad (TC-1000 Temperature Controller, CWE Inc., Ardmore, USA). The animals were tracheotomized and artificially ventilated (Rodent Ventilator 7025, Ugo Basile, Comerio, Italy). The heart rate and end-tidal CO_2_ concentration were continuously monitored by a custom ECG monitor (Otoconsult, Frankfurt a.M., Germany) and a capnometer (Normocap CO_2_ & O_2_ Monitor, Datex, Helsinki, Finland) respectively. The animals’ heads were fixed in a frame (Stereotaxic Frame 1430, David Kopf Instruments, Tujunga, USA) with a customized head holder. Using retroauricular incisions and dissection of tissue, the tympanic-bulla was uncovered to open the bone. The cochlea was partially exposed. A silver ball electrode was placed in the round window niche with contact to the round window membrane (RWM) (Fig. 5a) in order to measure CAPs. A cochleostomy was drilled in the *scala tympani* of the basal turn of the cochlea. The laser beam delivering optical fiber was placed through the cochleostomy inside the perilymph of the *scala tympani* (Fig. 5a) using a micromanipulator (MM33, Märzhäuser Wetzlar GmbH & Co. KG, Wetzlar, Germany). Hearing function was tested before and after surgery by measuring click-evoked auditory brainstem responses (ABRs, Butterworth filter 6^th^ order, high-pass frequency 200 Hz, low-pass frequency 5 kHz, amplification 100 dB) via subcutaneous Ag/AgCl electrodes (recording software: AudiologyLab, Otoconsult, Frankfurt a.M., Germany). Condensation clicks of increasing intensity were generated by a headphone speaker (DT48, Beyerdynamic, Heilbronn, Germany) directed at the animals’ outer ear at a ~3 cm distance. The ABR signals at each intensity level were averaged over 100 repetitions. Hearing was considered to be normal for response-thresholds at click-intensities corresponding to peak sound-pressure-levels of 20–30 dB_SPL_. During the experiments CAPs were recorded from the ball-electrode at the round window. The signal was band-filtered (Butterworth filter 6^th^ order, high-pass frequency 5 Hz, low-pass frequency 5 kHz) and amplified by 80 dB. Additional information of the CAP amplitude in dependence of the optical fiber position can be found as Supplementary Fig. S1.

### Analogous optoacoustic measurement

For the investigation of the signal generation mechanism a hydrophone (8103, Brüel & Kjær Sound & Vibration Measurement A/S, Denmark) was used to measure the pressure-changes at the fiber tip. The hydrophone was horizontally mounted inside a water-filled acrylic glass cylinder (height: 194 mm, diameter: 194 mm, volume: 5.7 l) and positioned next to the optical glass fiber (Fig. 5b). The dimension of the cochlea and the cylinder were very different (the guinea pig cochlea with an overall fluid volume of ~10 μl^45^ compared to a volume of 5.7 l for the cylinder used in the experiments), thus the physical modelling does not include any acoustic resonant features of the cochlea. The distance between the hydrophone and fiber was set at 0.1 mm using a three-axis translation stage with micrometers (XYZ Translation Stage, Thorlabs, NJ, USA). The signal was amplified with a signal conditioner and amplifier (Nexus 2692-0S1 bandwidth of 100 kHz, Brüel & Kjær Sound & Vibration Measurement A/S, Denmark). The recordings where averaged over ten thousand laser pulses to improve the signal-to-noise ratio (SNR). The laser system and the hydrophone data acquisition where synchronized via commercial software (Polytec, Waldbronn, Germany). The cylinder was filled with water. Water was used instead of perilymph for hydrophone measurements, because water is the main component of perilymph^43^ and also the main absorber in the perilymph in the near infrared region. Especially, as the proteins of the perilymph absorb mainly in the UV range^46^. To adjust the absorption coefficient, India ink (Royal Talens, Apeldoorn, Netherlands) was added to the water, producing an aqueous solution of different ink concentrations (0.001%; 0.005%; 0.01%; 0.05%; 0.1%; 0.5%; 1.0%; 5.0%). The optical transmission of the concentrations was measured using a spectrometer (wavelength range: 175–3300 nm, Lambda 900 spectrometer, Perkin Elmer, MA, USA) and the absorption coefficient was calculated according to Lambert-Beer’s law. For measurements with black India ink the wavelength was constant at 1300 nm (μ_α(1300 nm,H2O)_ = 1.34 cm^−1^ 32,33), which is below the reversal point of the wavelength study of *Schultz et al.*^22^. The functional validity of the correlation between the physiological response and the absorption coefficient observed in previous *in vivo* studies was analyzed by recording the pressure amplitudes with a hydrophone inside the fluid-filled cylinder as a function of absorption coefficient.

### Stimulating lasers

In this study, two laser systems were used in order to cover the appropriate parameter range for analyzing the optical stimulation in thermal and stress confinement conditions. The first laser (Ekspla, NT342A, Vilnius, Lithuania) was a tunable system comprised of an optical parametric oscillator (OPO). The wavelength could be adjusted between 420 nm and 2300 nm. The pulse duration was fixed at 4 ns at a repetition rate of 10 Hz. The second laser (Capella, Lockheed Martin Aculight, WA, USA) had a variable pulse duration from 10 μs up to 20 ms with a fixed wavelength of 1860 nm, a wavelength prominently used for INS in literature^27^,^41^. The repetition rate (between 2.5–100 Hz) depended on the pulse duration. Both laser systems were coupled into a multimode optical fiber (FG105LCA-CUSTOM, 105 μm core diameter, NA 0.22, low OH, Thorlabs, NJ, USA) via a fiber coupling unit. At first the pulse duration was varied (5 ns–200 μs) at fixed pulse energy (6 μJ) and a wavelength of 1860 nm. The second set of experiments was performed with a constant pulse peak power of 150 mW and variable pulse durations between 10 μs up to 10000 μs. Thirdly, the wavelength was changed between 845 nm and 2080 nm with constant pulse energy of 6 μJ. The pulse peak power and the pulse energy were measured at the fiber tip in air with a pyroelectric energy detector (Gentec-EO, Quebec, Canada) or with a thermal power sensor (PS10Q Laser Power Meter, Coherent, CA, USA).

**Figure 6:**
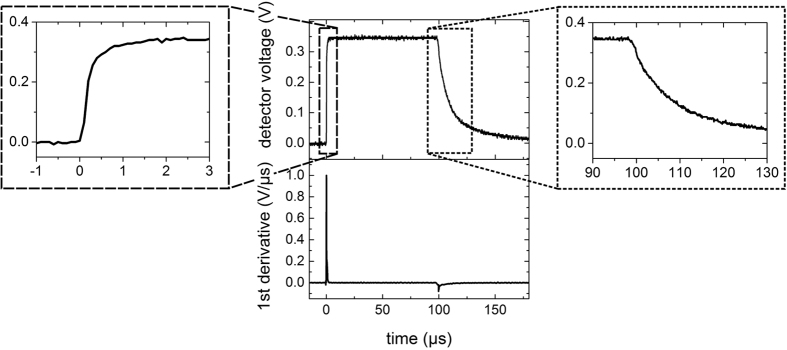
Temporal function of a 100 μs laser pulse measured by a photodiode (center top) and the first derivative of that laser pulse (center bottom). The insets show the pulse onset (left) and offset (right) on the expanded time axis.

The temporal laser pulse form (τ ≥ 10 μs) was detected using an InGaAs detector (DET10D, Thorlabs, NJ, USA) with a rise time of 25 ns (Fig. 6, top). The first derivative of the pulse power with respect to time was calculated numerically by averaging forward and backward derivative (Fig. 6, bottom).

Accordingly, water tank and *in vivo* measurements were performed with varying wavelengths, pulse durations, pulse energies, pulse peak powers and sample media with different absorption coefficients. In order to analyze the difference between stress and thermal confinement both laser systems were used. For pulses of 1860 nm delivered from a 105 μm fiber thermal confinement is fulfilled for pulse duration shorter than 4.9 ms (see equation (1), δ = 52.5 μm, κ = 1.4 * 10^−7^ m^2^/s). For wavelengths λ ≤ 2600 nm the pulse duration of the laser systems must be shorter than 35 ns to fulfill the stress confinement condition (see equation (2), 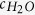 = 1484 m/s^47^).

## Additional Information

**How to cite this article**: Kallweit, N. *et al*. Optoacoustic effect is responsible for laser-induced cochlear responses. *Sci. Rep.* **6**, 28141; doi: 10.1038/srep28141 (2016).

## Supplementary Material

Supplementary Information
