# Supplementary material for: Optoacoustic effect is responsible for laser-induced cochlear responses
Source: Sci Rep. 2016 Jun 15;6:28141. doi: 10.1038/srep28141 (PMC4908384; doi:10.1038/srep28141)
Supplement: Supplementary Information [file srep28141-s1.pdf]

Supplementary Information

N. Kallweit[1,3,*], P. Baumhoff[2], A. Krueger[1,3], N. Tinne[1,3], A. Kral[2,3], T. Ripken[1,3], H. Maier[2,3]

[1]Laser Zentrum Hannover e.V., Hollerithallee 8, 30419 Hannover, Germany;

[2]Institute of Audioneurotechnology and Dept. of Experimental Otology, ENT Clinics, Hannover Medical School, Feodor-Lynen-Str. 35, 30625 Hannover, Germany;

[3]Cluster of Excellence "Hearing4all"

*n.kallweit@lzh.de

SUPPLEMENTARY FIGURE

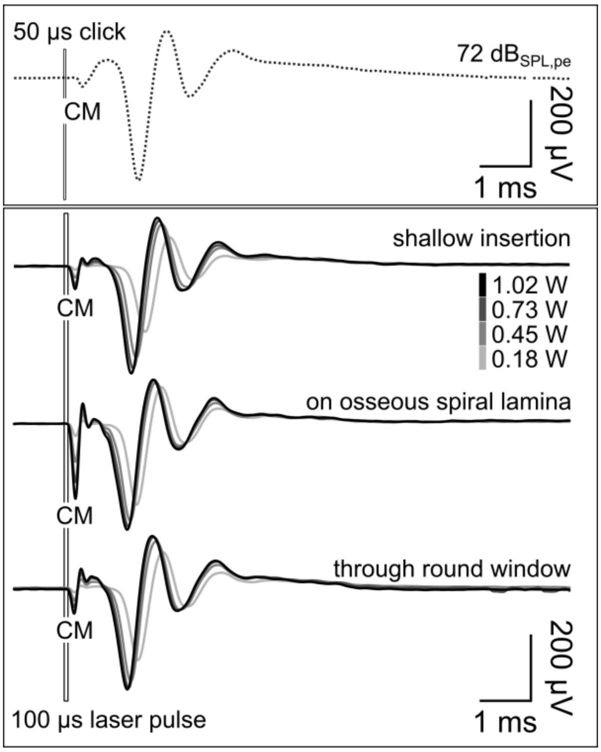

Supplementary Figure S1. CAP amplitude in dependence on three different fiber positions and compared to a click-evoked CAP. Laser pulse duration of 100 µs was tested with different pulse peak powers (1.02 W, 0.73 W, 0.45 W, 0.18 W). The fiber was placed firstly at the beginning of the cochleostomy (shallow insertion), secondly directly in contact with the osseous spiral lamina and thirdly resting on to the round window membrane pointing to the basilar membrane. This did not result in significant differences in the CAP amplitude. The cochlear microphonic potential (CM), commonly assumed to be generated by outer hair cell activity, is largest for a stimulation on the osseous spiral lamina. The presence of a CM component in the recorded signal for all fiber positions points to a mechanical origin of the activity. The grey vertical bar represents the time of the click (top) and laser irradiation (bottom).

.

SUPPLEMENTARY TABLE

Wavelength (nm)	Absorption coefficient (cm^{-1})
845	0.04
975	0.5
1000	0.4
1070	0.15
1190	1.25
1300	1.3
1370	5.45
1429	31
1550	12.1
1700	5.65
1850	10.8
1860	57.5
1927	120
1961	110
2100	31

Supplementary Table S1. The wavelengths and the associated absorption coefficients for water used for the wavelength study [32,33].
